# Variation in cross-sectional indicator of femoral robusticity in *Homo sapiens* and Neandertals

**DOI:** 10.1038/s41598-022-08405-8

**Published:** 2022-03-18

**Authors:** Anna Maria Kubicka, Antoine Balzeau, Jakub Kosicki, Wioletta Nowaczewska, Elżbieta Haduch, Anna Spinek, Janusz Piontek

**Affiliations:** 1 Department of Zoology, Poznań University of Life Sciences, Wojska Polskiego 28, 60-625 Poznan, Poland; 2 PaleoFED Team, UMR 7194, Département Homme et Environnement, CNRS, Muséum national d’Histoire Naturelle, Musée de l’Homme, 17, Place du Trocadéro, 75016 Paris, France; 3 Department of African Zoology, Royal Museum for Central Africa, 3080 Tervuren, Belgium; 4 Department of Avian Biology and Ecology, Adam Mickiewicz University in Poznań, Uniwersytet Poznański 6, 61-614 Poznan, Poland; 5 Department of Human Biology, University of Wrocław, Przybyszewskiego 63, 51-148 Wrocław, Poland; 6 Department of Anthropology, Jagiellonian University in Kraków, 31-034 Kraków, Poland; 7 Department of Anthropology, Hirszfeld Institute of Immunology and Experimental Therapy, Polish Academy of Sciences, Rudolfa Weigla 12, 53-114 Wrocław, Poland; 8 Institute of Human Evolutionary Biology, Adam Mickiewicz University in Poznań, Uniwersytet Poznański 6, 61-614 Poznan, Poland

**Keywords:** Anthropology, Anatomy

## Abstract

Variations in the cross-sectional properties of long bones are used to reconstruct the activity of human groups and differences in their respective habitual behaviors. Knowledge of what factors influence bone structure in *Homo sapiens* and Neandertals is still insufficient thus, this study investigated which biological and environmental variables influence variations in the femoral robusticity indicator of these two species. The sample consisted of 13 adult Neandertals from the Middle Paleolithic and 1959 adult individuals of *H. sapiens* ranging chronologically from the Upper Paleolithic to recent times. The femoral biomechanical properties were derived from the European data set, the subject literature, and new CT scans. The material was tested using a Mantel test and statistical models. In the models, the polar moment of area (J) was the dependent variable; sex, age, chronological period, type of lifestyle, percentage of the cortical area (%CA), the ratio of second moment areas of inertia about the X and Y axes (Ix/Iy), and maximum slope of the terrain were independent covariates. The Mantel tests revealed spatial autocorrelation of the femoral index in *H. sapiens* but not in Neandertals. A generalized additive mixed model showed that sex, %CA, Ix/Iy, chronological period, and terrain significantly influenced variation in the robusticity indicator of *H. sapiens* femora. A linear mixed model revealed that none of the analyzed variables correlated with the femoral robusticity indicator of Neandertals. We did not confirm that the gradual decline in the femoral robusticity indicator of *H. sapiens* from the Middle Paleolithic to recent times is related to the type of lifestyle; however, it may be associated with lower levels of mechanical loading during adolescence. The lack of correlation between the analysed variables and the indicator of femoral robusticity in Neandertals may suggest that they needed a different level of mechanical stimulus to produce a morphological response in the long bone than *H. sapiens*.

## Introduction

The structure of a bone is optimized in accordance with altered loading regimes, as manifested in the specific morphology, the orientation of the trabecular networks, and the size and distribution of the osteons^1^. However, the precise mechanism of mechanical signal detection is still investigated^1–3^ especially given that each bone differs in terms of sensitivity to mechanical stimuli^4^. Recently, most research has assessed limb bone organization using cross-sectional geometry, which is typically calculated from periosteal and endosteal contours of specific cross-sections^5–8^. Due to the broader availability of CT and the development of semiautomated techniques, interpopulation comparisons on larger scales can now be carried out. Thus, cross-sectional geometry is being used to measure diaphyseal variables which characterize bending and torsional rigidity^9^.

According to Macintosh and Stock ^10^, minimizing stress in cortical bone renders the postcranial skeleton (especially the lower limbs) highly reactive to changes in types and levels of mobility and activity patterns. This variability in bone morphology can be applied in paleontological and bioarchaeological research to reconstruct the lifestyles of past populations. Differences in the biomechanical properties of the lower limbs between preagricultural and agricultural populations suggest that sedentary societies may be characterized by greater size and rigidity in the long bones than highly mobile societies, possibly due to a greater variety of physical activity types in agricultural group^11^. However, despite a great deal of research analyzing variation in cross-sections, the effect of mobility levels on the quantity and distribution of cortical tissues in the femur exhibits discrepancies in terms of results^12^. For example, Carlson et al ^13^. showed that less mobile populations exhibited greater robusticity in the femoral midshaft than Australian aborigines. In contrast, research carried out on populations from the forest biome, and the fynbos of southern Africa revealed significant differences only in the femoral midshaft indices (i.e., ratio of maximum to minimum second moments of area) and between women^14^. Sládek et al. ^15^ claimed that a transition in mechanical loading between Late Eneolithic and Early Bronze age groups from Central Europe was present only in females and exhibited a mosaic pattern. In contrast, Friedl et al^16^. found no robusticity trend in lower limbs but rather a fluctuating pattern in Holocene populations. Interpretation of biomechanical changes may also be hampered, as terrain type (e.g., flat, hilly, mountainous) may influence variation in biomechanical properties of the lower limbs^17–21^. These results may indicate that geometric properties and loading patterns are not straightforwardly related and that should also consider additional biological and environmental factors in biomechanical analysis.

Some issues can be resolved by studying variations in cross-sectional properties of lower limbs in large samples^22^. Such analyses usually focus on populations from western Europe^19,22–24^, North America^12^, and Mesoamerica^25^. Studies on past human populations from eastern Europe, the Middle East, and Australia are very scarce^13,26^. The overrepresentation of certain geographical regions in biological studies entails two consequences. First, geographically proximate populations tend to be biologically similar; thus, certain variables such as overall body and craniofacial size may be spatially correlated^27^. In cases where samples are not equally numerous and evenly distributed in space, the results may be biased in favor of regions with more numerous samples. Therefore biomechanical properties of long bones should be checked for spatial dependence and, if necessary, analyses should be carried out following control for the effect of autocorrelation between populations. Secondly, new biomechanical data from Eastern Europe or the Middle East might clarify the local patterns of cross-sectional variables, which could be helpful in understanding the process of changes in global trends in postcranial morphology.

Studies on the cross-sectional geometry of the long bones are also used to analyze the differences between Neandertals and *H. sapiens*^6,28^. Previous research has shown greater diaphyseal robusticity in Neandertal lower limbs than in *H. sapiens*^29–32^. Variation in biomechanical properties of the upper and lower limbs is explained as the result of different levels of mechanical loading experienced throughout life^33,8^ or combinations of activities differing between Neandertals and *H. sapiens*^29^. However, the relationship between mechanical loads and level of robusticity is not straightforward due to the factors influencing the transmission of mechanical stimuli to bone structure, such as loading intensity, repetitiveness, number of loading cycles, or loading directions (principal or atypical)^34^. Research on differences in cross-sectional properties between *H. sapiens* and Neandertals assumes that the same level of mechanical loading causes a similar response of the biological mechanisms (i.e., growth, modeling, and remodeling of long bones)^35^; however, this aspect has not been analyzed yet. Therefore, we checked what factors can potentially play a significant role in shaping the femoral robusticity indicators, separately for Neandertals and *H. sapiens*. Since our research is not an experimental study, we will not investigate the cause–effect relationships between the femoral rigidity and variables. Nevertheless, we believe that the results will help understand the causes of variation in long bone cross-sectional geometry. Without understanding the nature of changes in bone rigidity and various factors, we will not be able to interpret the differences between those two hominin species correctly.

Previous studies analyzing differences in long bone geometry have usually focused on simple statistical analysis examining only a few variables at a time^36,37^. We decided to create a model that would take into account the chronological context and influence of biological (sex, age), environmental (terrain), and cultural (type of lifestyle) variables on the polar moment of area (J). The J index is considered a marker of femoral robusticity, as it characterizes resistance to the torsional and bending rigidity loads of the lower limb^38^. Based on previous research showing the variability in the femoral cross-section in hominins^38–42^, we predict that each factor will similarly correlate with the standardized index of femoral robusticity in Neandertals and *H. sapiens*. This analysis was carried out on an extensive database representing Neandertals and *H. sapiens* that have lived in Europe, Asia, and Australia from Paleolithic to modern times.

## Results

Descriptive statistics indicate that %CA is the least varied variable in *H. sapiens* and Neandertals. In turn, the standardized index of femoral robusticity (size-adjusted J), Ix/Iy, and age show a large variability in both hominin species (Tables S3, S4). Females and males of *H. sapiens* from the Middle Paleolithic are characterized by the highest means of size-adjusted J. The mean values of Neandertal females are slightly higher, while Neandertal males show slightly lower and similar means of size-adjusted J to *Homo sapiens* individuals from the Upper Paleolithic and Mesolithic. The mean relative amount of cortical area (%CA) in the femoral cross-sections of *H. sapiens* and Neandertals varied from 70.39 to 85.27%. The highest means of Ix/Iy were observed in *H. sapiens* from the Middle Paleolithic. The ratio of the second moment area of inertia about the X and Y axes (Ix/Iy) of Neandertals was close to 1. The differences in mean age between sexes, though not pronounced, varied between chronological periods.

**Figure 1:**
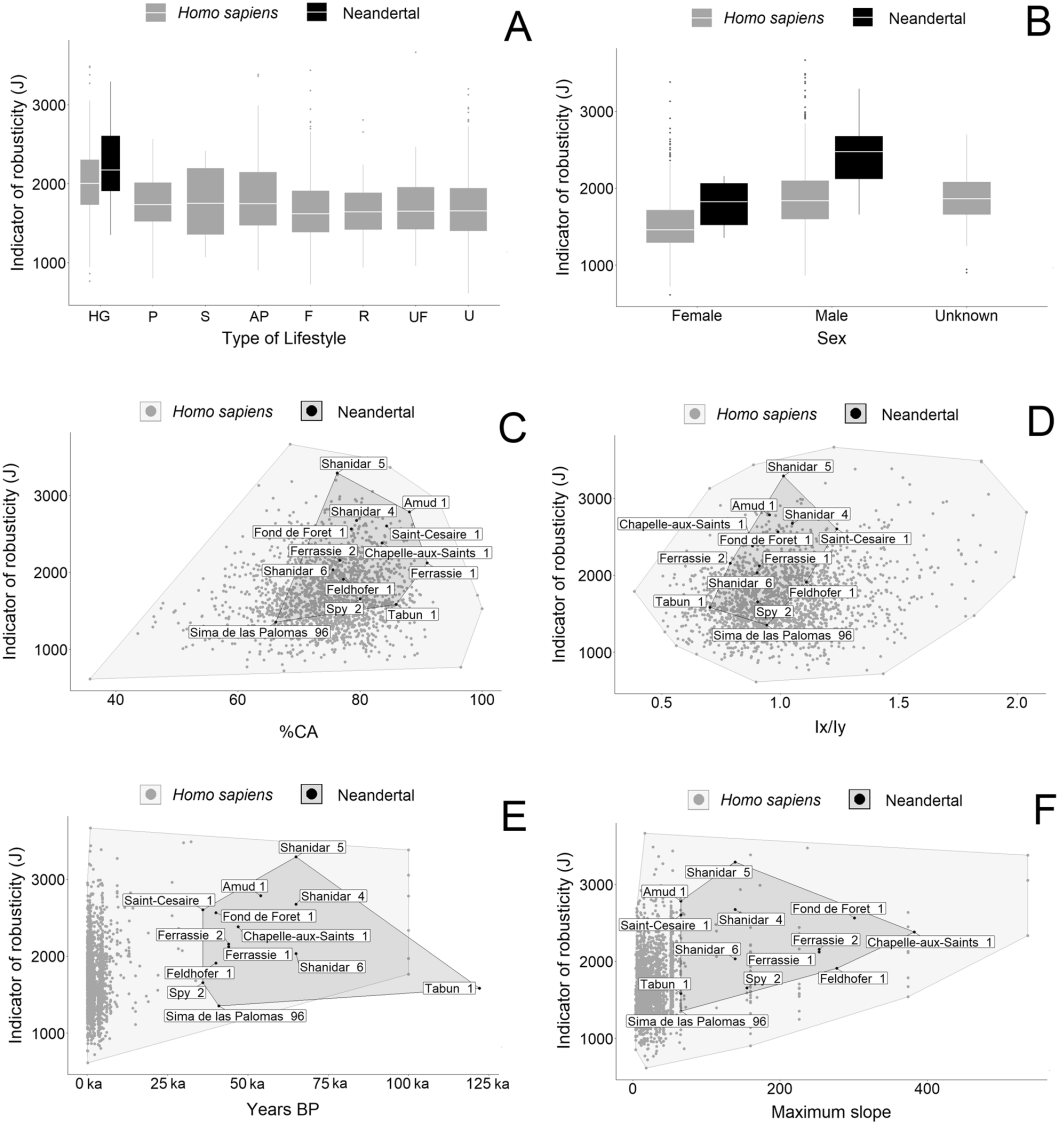
A graphic representation of the association between the standardized indicator of robusticity (size-adjusted J) and analyzed predictors in a cross-section of the femur. (**A**) Standardized indicator of robusticity (size-adjusted J) according to type of lifestyle. (**B**) Standardized indicator of robusticity (size-adjusted J) according to sex. (**C**) Association between standardized indicator of robusticity (size-adjusted J) and %CA. (**D**) Association between standardized indicator of robusticity (size-adjusted J) and Ix/Iy. (**E**) Distribution of standardized indicator of robusticity (size-adjusted J) during the chronological periods. (**F**) Association between standardized indicator of robusticity (size-adjusted J) and maximum slope of the terrain. *HG* hunting-gathering, *P* pastoralism, *S* seminomadic, *AP* agropastoralism, *F* farming, *R* rural, *UF* urban/farming, *U* urban.

The Mantel test revealed that the index of femoral robusticity (size-adjusted J) is spatially dependent in *H. sapiens* but not in Neandertals. Therefore, a generalized additive mixed model (GAMM) that removed the autocorrelation effect was used to investigate the variation in the *H. sapiens* sample. The GAMM showed that variables such as the chronological period in years (BP), sex, %CA, Ix/Iy, and maximum slope of the terrain significantly correlated with the standardized index of femoral robusticity (size-adjusted J) in *H. sapiens* (Table 1, S5). In the case of a linear mixed model (LMM), none of the analyzed predictors (years, maximum slope, sex, %CA, or Ix/Iy) significantly influenced the standardized indicator of femoral robusticity (size-adjusted J) in Neandertals (Table 2, S5). Figure 1A shows that different types of lifestyle observed in the *H. sapiens* sample do not differ in the level of the femoral robusticity indicator (size-adjusted J). Males of *H. sapiens* show higher dependent variable levels (size-adjusted J) than females, but with values close to the individuals of unknown sex (Fig. 1B). In the case of Neandertals, the differences in the femoral robusticity indicator (size-adjusted J) between sexes are visible but not statistically significant (Fig. 1B). According to the GAMM model and Fig. 1C,D, the femoral robusticity indicator (size-adjusted J) in *H. sapiens* increases simultaneously with two predictors (i.e., %CA and Ix/Iy). In the case of the *H. sapiens* sample, there is a decline in the robusticity indicator (size-adjusted J) over time, while no trend appears for the Neandertals (Fig. 1E). The maximum slope of terrain also increases with the femoral robusticity indicator but is only visible in the *H. sapiens* sample and not in the Neandertals (1F).

**Table 1:** Results of the generalized additive mixed model (GAMM) calculated for Homo sapiens^a^.

Effect	Estimate	SE	T	*p*
Intercept	2079.19	33.66	61.75	< 0.001
Years (BP)	12.71	13.41	3.49	**0.020**
Lifestyle	2.07	3.81	0.54	0.582
Sex	− 262.25	16.25	− 16.13	**< 0.001**
Age	35.67	19.79	1.80	0.071
%CA	36.80	32.83	10.25	**< 0.001**
Ix/Iy	125.99	28.68	4.39	**< 0.001**
Maximum slope	− 4.61	17.44	3.05	**0.041**

*SE* standard error, *T* value of the *t* test, *Age* mean age of each individual, *%CA* percent of cortical area in a cross section, *Ix/Iy* ratio of Ix and Iy, R^2^ = 0.229.

^a^Side of the body was used as a random effect with SD = 13.54. Bold indicates statistically significant at *p* < 0.05.

**Table 2:** Results of the linear mixed model (LMM) calculated for Neandertals^a^.

Effect	Value	SE	T	*p*
Intercept	1123.98	4437.10	0.25	0.800
Years (BP)	− 0.0012	0.012	-0.10	0.920
Sex	− 395.26	463.85	-0.85	0.420
%CA	12.41	29.25	0.42	0.680
Ix/Iy	804.46	1945.94	0.41	0.690
Maximum slope	− 0.534	2.24	− 0.23	0.820

*SE* standard error, *T* value of the *t* test, *%CA* percent of cortical area in a cross section, *Ix/Iy* ratio of Ix and Iy, R^2^ = 0.0054.

^a^Side of the body was used as a random effect with SD = 143.97. Bold indicates statistically significant *p* < 0.05.

## Discussion

### Spatial autocorrelation

The populations of *H. sapiens* are not evenly distributed in space; therefore, some geographical regions are over-represented by human groups. Our results do not clarify which factors among gene flow^43^, migration leading to a patchy distribution of a given variable^44,45^, a spatiotemporal pattern in the distribution of food, or various biotic and abiotic factors^46^ contributed the most to the spatial autocorrelations of femoral robusticity indicator (size-adjusted J) in *H. sapiens*. Most of the analyzed populations belonged to hunter-gatherer or nomadic societies and were from periods in which significant changes occurred in the structure and economy of populations. Therefore, it can be assumed that this spatial autocorrelation may result from two factors: migration events causing gene flow and the shifts in the amount of physical activity between the analyzed societies.

The Neandertal samples were not spatially dependent. It is difficult to characterize the chronospatial diversity of Neandertals due to an insufficiently large sample; however, the northern regions of Eurasia were settled by heavier and more robust individuals than those in South Europe^37,47,48^. Moreover, the fossils from Asia are dated from 122 to 54 ka; in turn, the European samples range from 47 to 36 ka. The observed lack of spatial autocorrelation can be explained by the overlapping of two variables: the large differences in chronology and the non-uniform distances between the sites^47^.

### Variation in the index of femoral robusticity

The high degree of variation in cross-sectional properties may be evidence of diverse ecology and subsistence demands in the analyzed *H. sapiens* groups. According to previous research^12,14,25,48–51^, the most important factors shaping hominin robusticity of the lower limbs are levels of mobility related to subsistence strategies, lifestyle, terrain, and climatic factors. Another study indicated a gradual decline in the cross-sectional properties of the lower limb during the Holocene due to intensification in food production^22^. Our study shows a slightly different relationship between the analyzed properties, factors such as years (BP), sex, two cross-sectional properties (%CA and Ix/Iy), and terrain have the greatest impact on the index of femoral robusticity (size-adjusted J).

Differences between females and males are more pronounced in the upper than in lower limbs^12,13,26,36,52^. Highly mobile groups are more sexually dimorphic than less mobile horticultural and industrial societies due to the greater role of the male in hunting activities compared to females^26,36^. However, Wescott^12^ emphasized that this kind of conclusion should be drawn based on the analysis carried out within subsistence strategies. The GAMM did not analyze whether mobile populations were more sexually dimorphic than sedentary groups; however, our results confirmed the previous research^25,26,15,53^ that sex was an important factor correlating with the level of femoral robusticity indicator (size-adjusted J). Apart from the amount of physical activity, other factors associated with the endocrine system, osteoporosis, genes, and development of the long bones may also be responsible for sex differences in the femoral rigidity^54–56^. Differences in the expansion period of the periosteal and endosteal surfaces cause the cortical bone mass in males to be placed farther from the neutral axis of the long bone cross-sections than it is in females^56^, thus explaining the greater bending rigidity in men than in women shown in our research.

Individuals of *H. sapiens* with greater bending rigidity in the anteroposterior plane (Ix/Iy > 1.0) were characterized by higher indexes of femoral robusticity (size-adjusted J) than samples with greater bending rigidity in the mediolateral plane (Ix/Iy < 1.0). The Ix/Iy ratio level reflects the variation in physical activity, as changes in levels of mobility may correlate with anteroposterior but not necessarily mediolateral bending^41^. If this assumption is accurate, we can expect that individuals of *H. sapiens* exposed to high mechanical loads (high index of size-adjusted J) and bending rigidity in the anteroposterior plane (Ix/Iy > 1.0) were also characterized by a wide variety of physical activity types of the lower limbs. Also, according to our analysis, the more robust the femur (size-adjusted J), the higher the content of cortical surface (%CA), which means that femurs more resistant to bending and with higher torsional rigidity, are better adapted to axial loads.

Populations occupying hilly and mountainous regions showed greater standardized indicators of femoral robusticity than populations from flat terrain^20,41^. We also found that *H. sapiens* inhabiting mountainous terrain exhibited more robust femora than individuals from lowlands. This association may partially explain why populations with the same lifestyle but inhabiting various terrain are characterized by different femoral biomechanical properties. It has also been suggested that rough terrain should exert the strongest influence on anteroposterior bending loads near the knee joint^18,19,41^. Perhaps that is why our model showed that greater degrees of femoral robusticity (size-adjusted J) were connected with increases in anteroposterior bending (values of Ix/Iy greater than 1.0).

Our statistical analysis also showed a decrease in the polar moment of area (size-adjusted *J*) in *H. sapiens* over the duration of chronological periods. Other research exhibited a fluctuating pattern rather than consistently observing decreasing or increasing trends^6,22,16^. Due to a lack of temporal decline in femoral head size and articular surfaces, Ruff et al.^38^ claimed that a decrease in mechanical loading on the postcranial skeleton, as opposed to genetic factors, was responsible for changes in femoral properties. In turn, Pearson^37^ suggested that fluctuations in bone rigidity were also a result of climate change. However, skeletal proportions are correlated with climate, and since the cross-sectional properties used here were standardized to the biomechanical length of the femur and body mass, we can expect that climate will not directly infleunce variation in femoral rigidity. On the other hand, climate influences environmental conditions, which can favor certain physical activity types (such as foraging) that require substantial mechanical power^37,57^. This means that climate may indirectly and partially influence their levels despite the standardization of the cross-sectional properties.

Lower levels of mechanical stimuli can be attributed to changes in food production associated with increased sedentism in European *H. sapiens* from the Upper Paleolithic to recent times^22,49^. Our results do not confirm the relationship between a more sedentary lifestyle and a decline in the femoral robusticity indicator of *H. sapiens*. Our assessment of the type of lifestyle may be prone to error since information on some human societies is still scarce, and foraging behavior is flexible due to short-term or local changes in the environment and food abundance. Other research on femoral rigidity and type of lifestyle has also failed to indicate any morphological pattern^12,22,49,58,59^. Stock and Pfeiffer^14^ drew different conclusions, as they found stronger lower limbs in a Later Stone Age African population than in a mobile marine group from the Andaman Islands. However, this research compared two populations from different chronological periods and geographical regions, influencing the results. The pattern between the index of femoral robusticity and type of lifestyle has not been revealed in this study, as the variation in physical activity levels among populations with the same type of lifestyle is large. Perhaps, the use of generalized categories typically for lifestyle is too broad even when analyzing large datasets.

Changes in diaphyseal rigidity begin to develop at an early stage of ontogeny as children improve their locomotor skills^33,60^ and may persist after a prolonged decline in physical activity levels^61^. In young females, the time of long-term loading before or after menarche is another crucial factor that regulates the reaction of the periosteal surface of the long bones to mechanical stimuli later in life^62^. Therefore, a gradual decline in the femoral rigidity observed in *H. sapiens* between the Middle Paleolithic to the present time may result from a different time of occurrence and lower physical activity levels before maturity. In addition, the increase in nutritional stress during the transition to agriculture may also contribute to a decline in postcranial robusticity^33^.

An increase in bone rigidity is observed until the fourth decade^16^ after which females show a decrease and males an increase in values of the biomechanical properties^63^. In our research, the variable age did not significantly correlate with the femoral robusticity indicator (size-adjusted J) of *H. sapiens*, which may be due to different age distribution and classes. Some individuals were assigned an average age of 38 years since the samples could not be classified more precisely in this regard. Perhaps, age is a relevant factor affecting changes in bone rigidity; however, for material comprising past populations, where the number of older adults (> 50 years old) is low, the aging trend may be ambiguous.

Values of size-adjusted J of the Neandertals fall within the range of the *H. sapiens* from the Mesolithic and Upper Paleolithic. However, it is difficult to indicate the group of *H. sapiens* to which two other Neandertal femoral properties (%CA and Ix/Iy) are most similar, as the relative cortical area and cross-sectional rigidity index showed no clear trend of changes in the former. The descriptive statistics showed that each Neandertal individual was characterized by a different level of torsional and bending rigidity loads of the lower limb. This variation may reflect an adaptation to physical activity experienced by Neandertal individuals during development^64^; however, it is still unclear how the level and type of physical activity may have differentially correlate with their femoral rigidity. Our results revealed that the variability in the standardized indicator of femoral robusticity in Neandertals was not caused by any of the investigated variables (i.e., chronological period, %CA, Ix/Iy, sex, or terrain). The coefficients of variation (CVs) of Neandertal variables are within the range of variation in human groups (Tables S3, S4), so we believe that the structure of their long bones was susceptible to changes to minimize the environmental impact pressure^65,66^.

Some research has revealed greater cross-sectional variables in Neandertals than in *H. sapiens*^8,50^; other research has found no differences in the level of robusticity between these two hominin species^29,32^. Nevertheless, differences in the femoral index and rigidity between Neandertals and *H. sapiens* are usually interpreted as differences in climatically-influenced types of habitual behaviors, hip mechanics, mechanical stress, or activity levels and patterning^32,16,64,67^. We showed that, whereas biological and environmental factors did not correlate with the variation in the femoral robusticity indicator (size-adjusted J) of Neandertals, some of them correlated with the femoral structures of *H. sapiens*. Perhaps those differences were not the level of environmental factors but the developmental plasticity of the postcranial skeleton.

The dental growth of Neandertals was accelerated relative to the dental development of *H. sapiens*^68,69^ (but see^70,71^). Still, the growth trajectory of Neandertal femora may appear delayed due to the accelerated dental development in Neandertals^72^. This slow femoral development was probably not directly affected by climate factors such as temperature or nutritional stress, given the lack of similarities in growth trajectory between Neandertals and the cold-adapted Inuit population^72^. The differences in skeletal morphology between Neandertals and *H. sapiens* at the beginning stage of postnatal development were probably caused by various genetic components^72–74^. The research on Neandertal locomotor efficiency shows that despite their shorter total lower limb length, no selective disadvantage in terms of locomotion on sloped terrain was found^75^. Moreover, an increase in the overall bending rigidity of the tibial midshaft is observed in Neandertals from non-flat terrain^21^. Therefore we suggest that the lower limbs of Neandertals are susceptible to bone modeling affected by mechanical loading but may need stronger stimuli than *H. sapiens* to cause changes in biomechanical properties. This can be supported by the concept of bone functional adaptation, assuming that bone tissue responds to mechanical stimuli, adjusting changes in its size, shape, and distribution^76^. According to Frost^35,77^, each bone has a threshold range at which bone strains above this threshold activate mechanically-controlled modeling as, e.g., bone resorption and deposition on the endosteal and periosteal surface. Perhaps, the level of mechanical loading was not high enough to generate modeling responses in the biomechanical properties of Neandertal long bones because the minimum effective signal may differ from that of *H. sapiens*.

Although all known Neandertal femurs for which body mass and biomechanical length were assessed, the sample size is small (but see other research^6,16,78^). Therefore, the ‘n effect’ was controlled in the analysis. Nevertheless, the possibility that non-significant predictors may be due to sample size effects should be taken into account so that new findings may better explain the changes in the biomechanical properties of Neandertal femora.

The research shows that factors such as terrain slope, level and type of physical activity, and mobility may have played an important role in shaping cross-sectional properties^21,34,41,15,60^. Statistical analysis on a large sample showed that variation in the femoral robusticity indicator of *H. sapiens* changed over time and was influenced by sex, terrain, and two biomechanical properties (%CA, Ix/Iy). In the case of the Neandertal samples, variation in femoral was not influenced by any of the analyzed factors. Since our research is not an experimental study that allows testing cause–effect relationships, it is difficult to identify precisely the reasons for the differences in the results between the samples of *H. sapiens* and Neandertals. Moreover, this cross-sectional study analyzes each individual at only a one-time point, not longitudinally. Nevertheless, based on the differences in the growth trajectories between *H*. *sapiens* and Neandertals^69–72^, we suggest that depending on what stage of postnatal development the mechanical loading begins at, the response of long bone will vary in these two hominin species. Second, *H. sapiens* and Neandertals may differ in the set point for minimum effective strain stimulus that causes bone changes^35^ as measured by biomechanical properties. The midshaft of the tibia was more responsive to loading than the femur among living women^10^; while Neandertals and Upper Paleolithic *H. sapiens* from mountainous terrain exhibited greater overall anteroposterior and mediolateral relative bending rigidity in the tibial midshaft than individuals from mixed and flat terrain^21^. This indicates regional variability in the distribution of strain in the lower limb and within a particular bone^10^ (but see^79^), proving that a more accurate interpretation of behavior will be possible when analysis of the plasticity levels of long bones has been conducted. Therefore, future research should focus not only on other elements of the postcranial skeleton but also on examining the level of bone response to different degrees of stimuli. The results may help us understand the differences in diaphyseal rigidity between hominin species and explain the causes of variability in robusticity within an individual.

## Summary

Our research shows that a lot of data on biological variables needs to be tested for spatial autocorrelation to avoid possible bias. Statistical analysis of the biomechanical properties of the lower limbs confirms previous studies, which showed a significant effect of variables such as sex, chronological periods, and terrain slope on femoral robusticity indicator. The decline in the diaphyseal rigidity of *H. sapiens* over certain chronological periods was not related to the type of lifestyle, as no difference in the indicator of robusticity was found between farmers, hunter-gatherers, pastoralists, seminomads, rural and urban societies. Perhaps this decline in postcranial robusticity was caused by a lower level of long-term loading during adolescence and an increase in nutritional stress during the transition to agriculture. Our analysis also clarified a positive correlation between the femoral robusticity indicator and two other biomechanical properties (%CA and Ix/Iy). Increases in %CA and anteroposterior rigidity (Ix/Iy > 1.0) may have helped the femur adjust to high mechanical loads during the life of an *H. sapiens* individual. The LMM showed that biological and environmental factors did not correlate with variation in the femoral robusticity indicator of Neandertals. This finding is surprising and may suggest the differences in the femoral response to mechanical loading between these two hominin species. As this research is not an experimental study, it is not possible to precisely indicate what levels of the analyzed variables are needed to obtain the effect of changes in biomechanical properties. However, based on the results, we suggest that the Neandertal femur required a different level of mechanical stimulus to produce a morphological response in the long bone than *H. sapiens*. The development of the Neandertal femur was delayed compared to that of *H. sapiens*, and possibly the level of mechanical stimuli was too low to produce a morphological response in Neandertal long bones. Assuming the lower limbs of *H. sapiens* were more susceptible to external factors than Neandertals, conclusions based on a comparative analysis between these two hominin species should be drawn with caution. The use of models to investigate the biomechanical properties of long bones enables the consideration of more variables and enhances the study of biological variability.

## Material and methods

The biomechanical properties of the femur used in this study were derived from three different sources: (1) the European data set, (2) new CT images of various osteological materials, and (3) the subject literature. Each type of material was described separately to clarify our methodological approach. The final database contained cross-sectional properties of 1972 individuals, of which 1750, 196, and 26 were derived from the European data set, new scans, and literature, respectively.

### Material from the European data set

The European data set was created as part of a project financed by the US National Science Foundation and carried out between 2007 and 2015. Within this project, a large amount of representative material, including many European populations from several major environmental transitions, was collected^41^. The database thus created contains the following variables for 2177 individuals: date range, geographic region, culture, site, geographical coordinates of the site, characteristics of the relevant terrain, sex, age range, linear dimensions of the postcranial skeleton, estimated anatomical stature and body mass, and cross-sectional dimensions of humeri, femora, and tibiae (for a detailed description of variables, see^41^). Biomechanical properties and other variables of all samples included in the European data set were collected using the same methodological approach^41^.

Cross-sectional properties in the European data set were calculated for long bones such as the humerus (1578 individuals), femur (1830 individuals), and tibia (1652 individuals). In the presented study, only femoral biomechanical properties from the European data set were analyzed; thus, data for further analysis contained 1830 individuals. Due to the low level of directional asymmetry of the lower limbs^80^, femoral biomechanical properties were selected from each individual from only one side of the body. Information about the side of the body was coded and used in the statistical models as a random factor. Since long bone diaphyseal rigidity is closely related to body size, some of the biomechanical properties of the weight-bearing lower limbs (e.g., J) had to be standardized to body mass and biomechanical length^81^. Therefore, individuals without estimated body mass and measured biomechanical femoral length were removed from the database. Of 1830 individuals, the amended data set consisted of 1750 individuals from the Early Upper Paleolithic (32,000–26,000 BP) to very recent times (after 1900 AD), aged between 15 and 87 years and from almost all of Europe^41^.

Furthermore, additional variables, such as the type of subsistence economy, were added to the amended data. Each individual or population was assigned one of the following types of lifestyle: hunting-gathering, farming, agropastoralism, pastoralism, seminomadic, rural, urban, urban/farming. This type of variable was added because each type of lifestyle is associated with a slightly different pattern of activity that influences bone development and formation during life^11,13^. In our opinion, biomechanical analysis in the context of lifestyle will expand knowledge of past populations and provide insights into behavioral diversity. While lifestyle assessment for some populations may be problematic, this was done based on archaeological and ethnographic literature, which allowed a complex view. Human societies that relied on hunting wild animals and foraging plants (e.g., fruits, vegetables, nuts) were classified as hunter-gatherers. The farming category was assigned to settled populations where domesticated crops (especially cereals and pulses) was the primary economic activity, while partially occupied human groups that relied on plant cultivation and livestock raising were classified as agropastoralists. Populations whose main economic activity was based on domesticated livestock such as goats, chickens, yaks, camels, sheep, and cattle were assigned to pastoralism. Seminomadic groups were defined as those for which evidence of seasonal migration and crop cultivation during periods of settlement was found. The rural category was assigned to communities living in nonurban areas where was a low ratio of inhabitants and the most important economic activities were usually agriculture and production of raw materials, but unlike farming societies, their settlements were close to urban centers. The urban category included heterogeneous groups in terms of socioeconomic status, living in densely populated settlements, where the most inhabitants have nonagricultural activities such as handicrafts and trade. The last lifestyle category, urban/farming, was assigned to populations growing crops (and sometimes also livestock) in towns and other urban areas. Table S1 contains the detailed list of human populations divided into archaeological/historical periods and geographical regions.

### Scanned material

To expand the database, femora from Neolithic populations from Poland (Bronocice, Kazimierza Mała, Koniusza, Samborzec, Smroków, Złota), modern Australian aborigines, and a mediaeval society from Poland (Ostrów Lednicki) were scanned in the cranio-caudal direction using cone beam computed tomography (CBCT) technique with the standard protocol (0.625 × 0.625 × 0.625 mm^3^) at a power level of 120 kV. All femurs were scanned using Fidex-FS CT system at the University Center of Veterinary Medicine (Poznań University of Life Sciences). The lower limbs of individuals from three Iranian populations (Dinkha Tepe, Hasanlu, and Tepe Hissar) were scanned by The Open Research Scan Archive using a CT with the same standard protocol (0.625 × 0.625 × 0.625 mm^3^). Based on the subject literature, for each new scanned individual, the same variables were collected as in the European data set: date range, geographic region, culture, site, and geographical coordinates of the site. Characteristics of the terrain were calculated within a 10 km radius of each archaeological site using QGIS software. According to Holt et al.^19^, a 10 km radius covers the terrain of a single-day foraging trip.

The material consisted of femora dated from 4950 to 3581 BP, derived from Tepe Hissar, located in the north-eastern Central Iranian Plateau. Excavations conducted by the University of Pennsylvania Museum show this settlement was occupied constantly from the fifth to the second millennium BC. The archaeological data suggest that the subsistence economy was based mainly on agriculture; thus, the investigated population was assessed as having been farmers^82^. Individuals from the Złota population originated in a Neolithic cemetery, dated from 4450 to 4180 BP, and located in a small Polish village near the river Żyć^83^. The instability and small size of the settlements, the chronological sequence of their development, and the great importance of animal husbandry in the economy suggest that this population was partially nomadic but also participated in agriculture and raising livestock^84,85^. Individuals from these human groups were assessed as agropastoralists. The remaining Polish Neolithic populations (Bronocice, Kazimierza Mała, Koniusza, Samborzec, and Smroków), dated to the period from 4210 to 3910 BP, inhabited the Little Poland Upland. Anthropological and archaeological literature indicates a partly nomadic lifestyle, including plant cultivation and breeding of livestock^84,86^; therefore, they were also classified as agropastoralists.

Moreover, the osteological material of two populations from West Iran was investigated. Dinkha Tepe (3252–3096 BP) and Hasanlu (3550–2350 BP) were settlements based on farming, located in fertile valley areas^86^. Another group derived from a mediaeval cemetery located in Ostrów Lednicki (Western Poland) dated from the mid-11th to the end of the fourth century AD^87^. This was a homogenous population in terms of socioeconomic structure; it relied mainly on agriculture but occasionally bred domestic animals (see Kubicka et al.^87^). Therefore, it was classified as a group of farmers. In the research, the femoral bones of Australian aborigines were also scanned. These individuals belonged to a modern hunter-gatherer society dated to the end of the nineteenth century AD, from West and South Australia^88^.

Skeletal remains accepted for the study were free of observable pathological changes (e.g., fractures, osteophytes, porosity) and free of visible wear on the periosteal surface of the shaft. The intact surface of the shaft is important because accurate modeling of the periosteal surface enables a high level of accuracy in the calculation of cross-sectional geometric properties^9^, especially since the total area is closely correlated with the polar moment of area, which is used to estimated diaphyseal rigidity^9^. The sex and age of the human populations from Australia, Ostrów Lednicki, Bronocice, Kazimierza Mała, Koniusza, Samborzec, Smroków, and Złota were assessed based on the standards of Buikstra and Ubelaker^89^ and White and Folkens^90^, using pelvis and skull (morphological traits and cranial suture closure). The individuals from Iran were classified as unknown sex due to the lack of bones other than femora that might have been used to assess them. The age status of these individuals was defined based on the maturation stages of the distal femoral end, femoral head, greater trochanter and lesser trochanter, using the standards of Buikstra and Ubelaker^89^ for the timing of the fusion of various postcranial elements. Individuals with a fully fused greater trochanter, lesser trochanter and a united (i.e. almost fused) femoral head and distal end were assessed as adults and included in the research. A more precise classification in terms of age was not possible, so that all individuals from the Iranian populations were assigned a mean age of 38 years. A similar solution was applied by Ruff^41^, who assigned all individuals lacking diagnostic elements an age of 38 years, which was the mean age for the entire European data set.

According to Auerbach and Ruff^91^, body mass was estimated with the use of mean values calculated from three formulas^92–94^, using femoral head superoinferior breadth. The biomechanical length of the femur was measured on CT images as the distance between a line tangential to the distal surfaces of the medial and lateral condyles and the deepest point on the superior surface of the femoral neck^95^. Next, an additional variable, such as lifestyle type, was added for each analyzed population, based on anthropological and archaeological literature. The newly scanned data consisted of 196 right femora of adult individuals (over 20 years old) from 10 different human populations from eastern Europe, western Asia, and Australia (see details in Table S1, S2). In cases where the right femur was poorly preserved, the left was included in the study. The femurs have been used with permission from museums where the osteological material is stored (the Museum of the First Piasts at Lednica, the University of Pennsylvania Museum of Archaeology and Anthropology, the Open Research Scan Archive at Penn). No administrative and ethical permissions were obtained because (1) according to Polish law there is no need to obtain permission from a biological commission when research is carried out on historical osteological collections, (2) no new archaeological excavations were carried out to gain the osteological material, (3) the research protocol used in this study was already tested by other researchers. The authors confirm that all stages of the research (collection, CT scanning and analysis) were performed in accordance with the regulations on the analysis of human remains.

### Material from the literature

According to the bioarchaeological literature, the femoral biomechanical properties of 13 adult Neandertals belonging to presumably 4 females (La Ferrassie 2, Sima de las Palomas 96, Shanidar 6, Tabun 1), and 9 males (Saint-Cesaire 1, Spy 2, La Ferrassie 1, Feldhofer 1, Fond-de-Forêt 1, Chapelle-aux-Saints 1, Amud 1, Shanidar 4 and 5) were collected^50,96–100^. Similar data from the literature were found for *H. sapiens* from the Upper and Middle Paleolithic. Tables S1 and S2 contain detailed lists of individuals. According to the methodology described by Ruff^41^ for the European data set, the following variables were collected for each individual: chronological date range, geographic region, coordinates of the site, archaeological site, sex, age range, biomechanical length of the femur, estimated anatomical stature and body mass, and raw cross-sectional dimensions of the femur. The characteristics of the terrain were calculated using QGIS software based on the geographical coordinates of the archaeological sites.

### Femoral biomechanical properties

The biomechanical geometric properties of the femora being scanned for the first time were calculated using the methodology proposed by Ruff^41^. The long bones were positioned according to a procedure developed by Ruff and Hayes^101^. A cross-sectional image was obtained using OsiriX software, for which the biomechanical geometric properties were then calculated using NIH ImageJ software with the Moment Macro developed by Ruff^102^. For each CT scan of the femur, a cross-section was created in the transverse plane, at 35% of biomechanical length as measured from the distal end of the bone. We focused only on the 35% cross-section, since this region is one of the most frequently analyzed (based on the European Data Set and paleoanthropological literature), and due to the massiveness of the distal femoral epiphysis, the preservation of this part is usually very good with no damage to the outer surface. For each 35% cross-section, five variables were determined: cortical area (CA), total area (TA), polar moment of area (J), and second moment areas of inertia about the X and Y axes (Ix and Iy, respectively, Table 3). Cortical and total areas were used to calculate the relative cortical area in a cross-section (Table 3). Ix and Iy were used to obtain the ratio of second moment areas of inertia about the X and Y axes, describing in which plane (anteroposterior or mediolateral) bending rigidity was greater (Table 3). We chose biomechanical geometric properties that are reported routinely in the subject literature in order to compare our findings with other research. Moreover, the calculated properties enable characterization of the axial, torsional, and bending rigidities of the lower limb. Various methods of standardization of biomechanical properties are still being debated; however, we used the procedure described in Ruff^41^, where the polar moment of area (J) was standardized to the estimated body mass and biomechanical length of the femur. Previous studies^22,40,16,103^ show that this standardized procedure of cross-sectional geometric properties is prone to errors. This aspect is important, especially in the Neandertal samples that are in a different state of preservation. Moreover, this standardization method is widely used and accepted by other paleoanthropologists; therefore, the new calculated values can be compared with previous results in further research. Further statistical analyses and interpretation of the results were carried out for the standardized indicator of femoral robusticity (size-adjusted J). The descriptive statictis are available in the supplementary material (Tables S3, S4).

**Table 3:** Description of variables used in the models.

Variable	Description
**Dependent variable**	
J (mm^4^)	Polar moment of area; describes resistance to torsional and bending rigidity loads. The variable is used as an indicator of robusticity; therefore, this variable was accepted in this research as an equivalent of femoral robusticity
**Independent variables**	
%CA	Relative amount of cortical area in a cross section, reflecting distribution of cortical area vs subperiosteal area as well as the resistance of the shaft to axial loadings. Calculated as: %CA = (CA/TA) × 100%, where CA is cortical area and TA is total area
Ix/Iy	Ratio of Ix (second moment area of inertia about the X axis) and Iy (second moment area of inertia about the Y axis), describing relative and bending rigidity in the anteroposterior plane relative to the mediolateral plane. Values close to 1 indicate equivalent rigidity in these two planes
Years (BP)	Age, calculated as the average of the period range
Lifestyle	Each population was classified as fitting one of the following types of economy: hunting-gathering, pastoralism, seminomadism, agropastoralism, farming, rural, urban/farming, urban
Maximum slope	Maximum slope of the terrain calculated within a 10 km radius of each archaeological site
Sex	Each individual was assigned to one of three categories: female, male, or unknown
Age	The mean age calculated for each individual as the average of the age range

### Statistical analysis

The observed variable of a population may be spatially dependent, meaning that its value depends on the variable of a neighboring group. Many analyzed variables are spatially correlated for various reasons, such as gene flow or natural selection resulting from similar environments^27,43,104^. For example, spatial autocorrelation analysis has revealed variation in human cranial variables^44^. Notwithstanding, studies of the relationship between biological and geographical distances have rarely been applied in biological anthropology^27^. In anthropological research, a component of spatial pattern should be an important factor, especially in considerations where many populations are examined, and some geographical regions are underrepresented.

Therefore, firstly, spatial autocorrelation for the dependent variable, i.e. the standardized indicator of robusticity (size-adjusted J), was tested separately for *H. sapiens* (n = 1959) and Neandertals (n = 13). For this purpose, we used Mantel tests^105^, which indicated whether the data were spatially dependent or not. To test the statistical significance of the Mantel statistics, Monte Carlo permutations with 999 randomizations were calculated^106^. According to this procedure, close spatial autocorrelation was found for the standardized indicator of femoral robusticity in *H. sapiens* (Mantel test: r_M_ = 0.062, *p* = 0.001), whereas for the femoral variable representing Neandertals, the Mantel test was not significant (r_M_ = 0.105, *p* = 0.105).

Before calculating the models for *H. sapiens* and Neandertals, two variables (age and years in BP) were calculated for each individual as the average of their range (Table 3). Next, a generalized additive mixed model (GAMM) was used in this study to analyze the effect of several variables on the standardized indicator of femoral robusticity (size-adjusted J) of *H. sapiens*. The GAMM was an extension of a general additive model enabling the inclusion of random factor and correction in spatial autocorrelation in the model. Therefore, as a response variable, we used the standardized indicator of robusticity (size-adjusted J), whereas years (in BP), type of lifestyle, maximum slope, sex, age, %CA, and Ix/Iy were used as predictors. Due to changes in relative bone rigidity over lifespans^16^, we decided to add age as a predictor to the GAMM. Differences in hormone levels between females and males also influence bone rigidity and the amount of cortical bone; accordingly, sex was also added to the model^107,108^. As far as we know, no other study has analyzed the relationship between J and Ix/Iy, and only one study has focused on the possible relationship between J and %CA in femurs^109^, which means that femoral cross-sectional properties have usually been investigated using separate statistical analyzes. According to Stock and Pfeiffer^14^, the shafts and epiphyses differ in plasticity. Moreover, the cross-sectional properties (e.g., %CA or Ix/Iy) indicate differences in developmental patterns among femoral shafts during successive locomotor stages and maturation^60^. Therefore, in our opinion, investigation of how other cross-sectional properties influence femoral robusticity based on a large dataset can provide valuable information about femur biomechanics. Furthermore, the side of the body was used as a random factor, along with the longitude and latitude of the archaeological sites as a spherical spatial correlation (Table 3).

Since the standardized indicator of the femoral robusticity of Neandertals was not spatially dependent, we used a linear mixed model (LMM) to analyze which factors influenced this parameter. The LMM was an extension of simple linear models, enabling the addition of random effects (e.g., side of the body). In this statistical analysis, we decided to use factors similar to those in the model with *H. sapiens* samples, except that we did not include two variables. The first excluded factor was the type of lifestyle since Neandertals represent exclusively a society of hunter-gatherers. Individual age was also excluded from the LMM due to the uncertain age assessment of some Neandertal specimens. In the LMM, the standardized indicator of femoral robusticity (size-adjusted J) was used as a response variable; years (in BP), maximum slope, sex, %CA, and Ix/Iy were used as predictors; side of the body was used as a random factor.

To allow some degree of complexity in the functions while avoiding overfitting of the data, we defined the basic dimension, i.e., k = 4^110^. The Gaussian distribution of errors and the identity link function were applied. As a measure of deviance reduction, we used R^2^^111^. The most parsimonious model was selected using the Akaike Information Criterion (Table S5, mgcv library in R^112^). Moreover, descriptive statistics such as group mean, median, standard deviation, variation, maximum, and minimum were calculated for all biomechanical properties (size-adjusted J, %CA, Ix/Iy) and mean age according to temporal period and sex. All statistical analyses were calculated using R software (R Core Team, 2017).

## Supplementary Information

Supplementary Information.

## Data Availability

The data is available on an external repository (Mendeley Data: 10.17632/zsnydxhhnf.1).
