# Publisher Correction: Variation in cross-sectional indicator of femoral robusticity in *Homo sapiens* and Neandertals

**DOI:** 10.1038/s41598-022-10446-y

**Published:** 2022-04-13

**Authors:** Anna Maria Kubicka, Antoine Balzeau, Jakub Kosicki, Wioletta Nowaczewska, Elżbieta Haduch, Anna Spinek, Janusz Piontek

**Affiliations:** 1 Department of Zoology, Poznań University of Life Sciences, Wojska Polskiego 28, 60‑625 Poznan, Poland; 2 PaleoFED Team, UMR 7194, Département Homme et Environnement, CNRS, Muséum National d’Histoire Naturelle, Musée de l’Homme, 17, Place du Trocadéro, 75016 Paris, France; 3 Department of African Zoology, Royal Museum for Central Africa, 3080 Tervuren, Belgium; 4 Department of Avian Biology and Ecology, Adam Mickiewicz University in Poznań, Uniwersytet Poznański 6, 61‑614 Poznan, Poland; 5 Department of Human Biology, University of Wrocław, Przybyszewskiego 63, 51‑148 Wrocław, Poland; 6 Department of Anthropology, Jagiellonian University in Kraków, 31‑034 Kraków, Poland; 7 Department of Anthropology, Hirszfeld Institute of Immunology and Experimental Therapy, Polish Academy of Sciences, Rudolfa Weigla 12, 53‑114 Wrocław, Poland; 8 Institute of Human Evolutionary Biology, Adam Mickiewicz University in Poznań, Uniwersytet Poznański 6, 61‑614 Poznan, Poland

Correction to: *Scientific Reports* https://doi.org/10.1038/s41598-022-08405-8, published online 18 March 2022

The original version of this Article contained an error in the link in the Data availability section where,

“The data is available on an external repository (Mendeley Data: https://doi.org/10.17632/fdpxmn5pjx.1).”

now reads:

“The data is available on an external repository (Mendeley Data: https://doi.org/10.17632/zsnydxhhnf.1).”

The original Article has been corrected.

