# Supplementary material for: Variation in cross-sectional indicator of femoral robusticity in Homo sapiens and Neandertals
Source: Sci Rep. 2022 Mar 18;12:4739. doi: 10.1038/s41598-022-08405-8 (PMC8933494; doi:10.1038/s41598-022-08405-8)
Supplement: Supplementary file 1 — Supplementary Information. [file 41598_2022_8405_MOESM1_ESM.doc]

**Supplementary Online Material (SOM):**

Variation in cross-sectional indicator of femoral robusticity in *Homo sapiens* and Neandertals

Anna Maria Kubicka ^a,b^, *, Antoine Balzeau ^b,c^, Jakub Kosicki ^d^, Wioletta Nowaczewska ^e^, Elżbieta Haduch ^f^, Anna Spinek ^g^, Janusz Piontek ^h^

^a^ *Poznań University of Life Sciences, Department of Zoology, 60-625 Poznań, Poland*

^b^ *PaleoFED team, UMR 7194, CNRS, Département Homme et Environnement, Muséum national d'Histoire naturelle. Musée de l’Homme, 17, Place du Trocadéro, 75016 Paris, France*

^c^ *Department of African Zoology, Royal Museum for Central Africa, 3080, Tervuren, Belgium*

^d^ *Adam Mickiewicz University in Poznań, Department of Avian Biology and Ecology, 61-614 Poznań, Poland*

^e^ *Wrocław University, Department of Human Biology, 51-148 Wrocław, Poland*

^f^ Jagiellonian University in Kraków, Department of Anthropology, 31-034 Kraków, Poland

^g^ *Polish Academy of Sciences, Hirszfeld Institute of Immunology and Experimental Therapy, Department of Anthropology, 53-114 Wrocław, Poland*

^h^ *Adam Mickiewicz University in Poznań, Institute of Human Evolutionary Biology, 61-614 Poznań, Poland*

* Corresponding author.

E-mail address: amkkubicka@gmail.com (A.M. Kubicka).

**SOM Table S1**

List of analyzed populations based on type of material and chronological period.^a^

| Type of data | Site | Date | Geographical area | Lifestyle economy | Number of individuals | | | |
| --- | --- | --- | --- | --- | --- | --- | --- | --- |
|  |  |  |  |  | Total | F | M | U |
| Middle Paleolithic | | | | | | | | |
| Literature | Amud, Chapelle-aux-Saints , Feldhofer, Ferrassie, Fond de Foret, Saint-Cesaire, Shanidar, Sima de las Palomas, Skhul, Spy, Tabun, Qafzeh | 122–36 ka | Asia, Europe | Hunting-gathering | 19 | 6 | 13 | 0 |
| Upper Paleolithic | | | | | | | | |
| European Data Set | Arene Candide, Barma Grande, Bichon, Bruniquel, Cap Blanc, Chancelade, Cro-Magnon, Dolní Vestonice, Grotte des Enfants, La Rochette, Neuessing, Oberkassel, Ostuni, Paglicci, Parabita (Veneri), Paviland, Riparo Continenza, Riparo Tagliente, Rochereil, Romanelli, Romito, Saint Germain-la-Rivière, San Teodoro, Sunghir, Villabruna | 28890–9875 BP | Europe | Hunting-gathering | 35 | 12 | 23 | 0 |
| Literature | Cro-Magnon, Minatogawa, Ohalo, Rochette | 30000–15000 BP | Asia, Europe | Hunting-gathering | 7 | 4 | 3 | 0 |
| Mesolithic | | | | | | | | |
| European Data Set | Birsmatten, Bottendorf, Culoz, Cuzoul de Gramat, Dragsholm, Gough's Cave, Hoedic, Holmegård, Koelbjerg, Le Rastel, Los Canes, Loschbour, Moita de Sebastiao, Molara, Mondeval, Muge Arruda, Schela Cladovei, Sejrø, Skateholm I, Skateholm II, Teviec, Uzzo, Vængesø 2, Vatte di Zambana | 9270–5160 BP | Europe | Hunting-gathering | 50 | 14 | 36 | 0 |
| Neolithic | | | | | | | | |
| European Data Set | A303, Ajvide, Åstrup Ås, Bad Durrenberg, Bad Sulza, Bedinge, Bilzingsleben, Bischleben, Bodal Mose, Borre, Braunsdorf, Brandysek, Brezno, Brozany, Bruchstedt, Dolni Vestonice, Døjringe, Drosa, Erfurt, Erfurt Nord, Erfurt-Nordhauser St., Fontenoce Recanati , Franzhausen I, Franzhausen II, Franzhausen IV, Franzhausen I (CWC), Franzhausen III (CWC), Frederiksberg, Fridtorp, Gemeinlebarn-Mitte, Gemeinlebarn F, Gjemild, Gjerrild, Großfahner, Großkayna, Haunø-Lauenkjor, Hallebyga°rd, Hazleton North, Hellested, Hjortholm Mose, Holubice IV, Ire, Kbely, Kelderød, Knezeves, Kobylisy, Kuclin, Langebjerg, Lanhill, Libesice, Lochenice, Mala ohrada, Marbjerg, Mokruvky, Most, Niederbosa, Obrnice, Orlishausen, Over Vindinge, Østrup Mose, Pavlov, Plotiste, Pohorelice, Pontcharaud, Poplze, Poregård, Postoloprty, Porsmose, Prosetice, Radegast, Radovesice, Rousi´nov, Schafstadt, Schlotheim, Schonstedt, Siroke Trebcice, Skendleby, Sondershausen, St.Tuborg, Store Lyng, Toedling, Tucapy, Tuchomerice, Udestedt, Unseburg, Västerbjers, Velika Ves, Veyrier, Viby, Vikletice, Viksø, Vrbice, Vyskov, Wayland's Smithy I, Weißensee, West Kennet, Zidovice | 7450–3650 BP | Europe | Farming, agropastoralists, pastoralism, hunting-gathering | 264 | 108 | 156 | 0 |
| New data | Bronocice, Kazimierza mała, Koniusza, Samborzec, Smroków, Tepe Hissar, Złota | 4950B–3096 BP | Asia, Europe | Farming, agropastoralism | 68 | 11 | 29 | 28 |
| Bronze | | | | | | | | |
| European Data Set | Amesbury, Bernhardsthal, Boscombe, Bystrocice, Castellon Alta (Galera 1), Castellon Alta (Galera 2), Castellon Alta (Granada), Chrastany 1, Cliff's End, Crichel Down, Franzhausen, Franzhausen I (U), Hainburg, Hulin-Pravcice 1, Gemeinlebarn F, Groszmugl, Hobersdorf, Melchendorf, Melk, Olmo di Nogara, Pitten, Pottenbrunn, Shrewton, Staxton, Stonehenge, Terrera Del Reloh, Unterhautzenthal, Wilsford S. Lake, Wuernitz | 4450–2650 BP | Europe | Farming, agropastoralism | 197 | 83 | 114 | 0 |
| New data | Dinkha Tepe, Hasanlu |  | Asia | Farming | 12 | 2 | 0 | 10 |
| Iron/Roman | | | | | | | | |
| European Data Set | Asnæs, Battlesbury Bowl, Bøgebjerg, Broskov, Cockey Down, Egebjerg, Endegårde, Englerup, Fraugde, Gammel Lundby, Græsbjerg, Halbturn, Himlingøje, Jenisuv Ujezd, Jinonice, Landlystvej, Lille Vasby, Lucus Feroniae, Lyregård, Makotrasy, Nordenbrogård, Næstved Mark, Øbjerggård, Poundbury, Poundbury Farm, Quadrella, Radovesice, Rue Jacques Brel, Sanderumgård, Scheelminde, Simonsborg, Store Grandløse, Store Keldbjerg, Varpelev, Vemmeltofte Skovridergår, Wetwang Slack | 1990–400 AD | Europe | Urban, rural, farming, | 270 | 131 | 139 | 0 |
| Early Medieval | | | | | | | | |
| European Data Set | Bruckneudorf, Jau Dignac, Laxenburger Str./Voesendorf, Mikulcice, Moedling, Roselle, Santa Maria de Hito, Vicenne Campochiaro, Villanueva de Soportiva, Zwentendorf | 5^th^–11^th^ century | Europe | Urban, rural, seminomadic, farming | 266 | 114 | 152 | 0 |
| Late Medieval | | | | | | | | |
| European Data Set | Blackgate, Dresden Briesnitz, Leiria, Mistihalj, Opava-Pivovar, Piazza della Signoria, San Baudelio de Berlanga, Sigtuna, Sporkova, Vratislavsky Palac, Vrsany, York, | 10^th^–16^th^ century | Europe | Urban, urban/farming, agropastoralism, pastoralism | 381 | 177 | 204 | 0 |
| New data | Ostrów Lednicki | 11^th^–14^th^ century | Europe | Farming | 92 | 40 | 36 | 16 |
| Early modern | | | | | | | | |
| European Data Set | Moirans, Observance, Porvoo, Renko, Siracusani, Spitalfields | 16^th^–19^th^ century | Europe | Urban, rural | 136 | 51 | 85 | 0 |
| New data | Australian aborigines | 19^th^ | Australia | Hunting-gathering | 24 | 4 | 17 | 3 |
| Very recent | | | | | | | | |
| European Data Set | Helsinki, Jena, Luis Lopez Collection, Sassari | 20^th^ century | Europe | Urban, farming | 151 | 57 | 94 | 0 |
|  |  |  |  | Total | 1972 | 814 | 1101 | 57 |

Abbreviations: F = female; M = Male; U = unknown sex.

^a^ ‘European Data Set’ from by Ruff (2018), ‘Literature’ denotes material collected from literature, ‘New data’ refers to material scanned and analyzed for the first time

**SOM Table S2**

Detailed list of populations added to the already existing European data set.

| Type of data | Site | Date | Geographical area | Lifestyle economy | Number of individuals | | | |
| --- | --- | --- | --- | --- | --- | --- | --- | --- |
|  |  |  |  |  | Total | F | M | U |
| Middle Paleolithic | | | | | | | | |
| Literature | Amud | 54 ka | Asia | Hunting-gathering | 1 | 0 | 1 | 0 |
|  | Chapelle-aux-Saints | 47ka | Europe | Hunting-gathering | 1 | 0 | 1 | 0 |
|  | Feldhofer | 40 ka | Europe | Hunting-gathering | 1 | 0 | 1 | 0 |
|  | Ferrassie | 44 ka | Europe | Hunting-gathering | 2 | 1 | 1 | 0 |
|  | Fond de Foret | 40 ka | Europe | Hunting-gathering | 1 | 0 | 1 | 0 |
|  | Saint-Cesaire | 36 ka | Europe | Hunting-gathering | 1 | 0 | 1 | 0 |
|  | Shanidar | 65 ka | Asia | Hunting-gathering | 3 | 1 | 2 | 0 |
|  | Sima de las Palomas | 41 ka | Europe | Hunting-gathering | 1 | 1 | 0 | 0 |
|  | Skhul | 100 ka | Asia | Hunting-gathering | 3 | 0 | 3 | 0 |
|  | Spy | 36 ka | Europe | Hunting-gathering | 1 | 0 | 1 | 0 |
|  | Tabun | 122 ka | Asia | Hunting-gathering | 1 | 1 | 0 | 0 |
|  | Qafzeh | 100 ka | Asia | Hunting-gathering | 3 | 2 | 1 | 0 |
| Upper Paleolithic | | | | | | | | |
| Literature | Cro-Magnon | 30 ka | Europe | Hunting-gathering | 1 | 0 | 1 | 0 |
|  | Minatogawa | 15 ka | Asia | Hunting-gathering | 4 | 3 | 1 | 0 |
|  | Ohalo | 19 ka | Asia | Hunting-gathering | 1 | 0 | 1 | 0 |
|  | Rochette | 22 ka | Europe | Hunting-gathering | 1 | 1 | 0 | 0 |
| Neolithic | | | | | | | | |
| New data | Bronocice | 4000 BP | Europe | Farming | 1 | 0 | 1 | 0 |
|  | Kazimierza mała | 3950 BP | Europe | Farming | 1 | 0 | 1 | 0 |
|  | Koniusza | 4000 BP | Europe | Farming | 2 | 0 | 2 | 0 |
|  | Samborzec | 4080 BP | Europe | Farming | 3 | 1 | 2 | 0 |
|  | Smroków | 3950 BP | Europe | Farming | 1 | 0 | 1 | 0 |
|  | Tepe Hissar | 4265 BP | Eurasia | Farming | 15 | 3 | 1 | 11 |
|  | Złota | 4315 BP | Europe | Agropastoralism | 45 | 7 | 21 | 17 |
| Bronze | | | | | | | | |
| New data | Dinkha Tepe | 3174 BP | Eurasia | Farming | 5 | 1 | 0 | 4 |
|  | Hasanlu | 2950 BP | Eurasia | Farming | 7 | 1 | 0 | 6 |
| Late Medieval | | | | | | | | |
| New data | Ostrów Lednicki | 11^th^ –14^th^ century | Europe | Farming | 92 | 40 | 36 | 16 |
| Early modern | | | | | | | | |
| New data | Australian aborigines | 19^th^ | Australia | Hunting-gathering | 24 | 4 | 17 | 3 |
|  |  |  |  | Total | 222 | 67 | 98 | 57 |

Abbreviations: Literature = material collected from literature; New data = scanned material and analyzed for the first time; F = female; M = Male; U = unknown sex

**SOM Table S3**

Descriptive statistics of the femoral biomechanical properties and age of *Homo sapiens* according to temporal period and sex

|  |  |  |  |  | Female | |  |  |  |  |  | Male |  |  |  |  |  |  | Unknown | |  |  |
| --- | --- | --- | --- | --- | --- | --- | --- | --- | --- | --- | --- | --- | --- | --- | --- | --- | --- | --- | --- | --- | --- | --- |
|  | Period | *n* | Median | Mean | Min | Max | CV | SD | *n* | Median | Mean | Min | Max | CV | SD | *n* | Median | Mean | Min | Max | CV | SD |
|  | J |  |  |  |  |  |  |  |  |  |  |  |  |  |  |  |  |  |  |  |  |  |
|  | Very recent | 57 | 1444.97 | 1481.86 | 771.53 | 2242.86 | 0.20 | 302.90 | 94 | 1778.86 | 1806.42 | 973.95 | 2950.47 | 0.18 | 324.17 |  |  |  |  |  |  |  |
|  | Early modern | 55 | 1382.92 | 1419.18 | 612.41 | 2035.08 | 0.22 | 307.24 | 102 | 1788.94 | 1804.37 | 863.33 | 2927.74 | 0.21 | 381.57 | 3 | 1940.75 | 1632.02 | 945.23 | 2010.07 | 0.36 | 585.78 |
|  | Late Medieval | 217 | 1565.27 | 1610.34 | 958.93 | 2777.79 | 0.21 | 331.89 | 240 | 1958.41 | 1962.16 | 1053.44 | 3664.77 | 0.19 | 370.30 | 16 | 1864.14 | 2005.23 | 1535.36 | 2697.65 | 0.18 | 362.02 |
|  | Early Medieval | 114 | 1402.68 | 1456.01 | 863.38 | 2289.81 | 0.20 | 288.98 | 152 | 1849.77 | 1886.76 | 1091.61 | 3436.48 | 0.19 | 365.59 |  |  |  |  |  |  |  |
|  | Iron/Roman | 131 | 1435.62 | 1456.35 | 721.58 | 3129.40 | 0.22 | 315.84 | 139 | 1716.27 | 1740.49 | 946.39 | 2964.19 | 0.22 | 398.34 |  |  |  |  |  |  |  |
|  | Bronze | 85 | 1321.10 | 1368.29 | 837.43 | 2907.80 | 0.24 | 323.92 | 114 | 1696.82 | 1724.46 | 962.58 | 2985.49 | 0.21 | 368.50 | 10 | 1950.03 | 1996.54 | 1527.62 | 2635.46 | 0.22 | 431.37 |
|  | Neolithic | 119 | 1516.88 | 1577.94 | 799.45 | 2606.72 | 0.23 | 363.35 | 184 | 1853.28 | 1908.14 | 1060.93 | 3384.09 | 0.21 | 410.08 | 29 | 1764.13 | 1813.43 | 904.61 | 2441.67 | 0.19 | 349.79 |
|  | Mesolithic | 14 | 1737.28 | 1732.07 | 1343.22 | 2307.11 | 0.19 | 325.35 | 36 | 2195.22 | 2185.47 | 1271.69 | 3272.62 | 0.21 | 448.81 |  |  |  |  |  |  |  |
|  | Upper Paleolithic | 16 | 1631.43 | 1719.26 | 1111.06 | 2525.99 | 0.26 | 450.24 | 26 | 2073.40 | 2206.64 | 1470.67 | 3488.71 | 0.24 | 536.17 |  |  |  |  |  |  |  |
|  | Middle Paleolithic | 2 | 2858.54 | 2858.54 | 2335.43 | 3381.66 | 0.26 | 739.79 | 3 | 2783.96 | 2534.49 | 1766.31 | 3053.22 | 0.27 | 678.76 | 1 | 1970.39 | 1970.39 | 1970.39 | 1970.39 | 0.00 | 0.00 |
|  | %CA |  |  |  |  |  |  |  |  |  |  |  |  |  |  |  |  |  |  |  |  |  |
|  | Very recent | 57 | 72.97 | 71.44 | 43.59 | 85.58 | 0.11 | 7.71 | 94 | 74.33 | 73.23 | 51.19 | 85.86 | 0.09 | 6.47 |  |  |  |  |  |  |  |
|  | Early modern | 55 | 71.01 | 70.39 | 35.74 | 96.48 | 0.15 | 10.25 | 102 | 73.23 | 73.65 | 57.77 | 87.31 | 0.09 | 6.43 | 3 | 83.76 | 85.27 | 82.66 | 89.39 | 0.04 | 3.61 |
|  | Late Medieval | 217 | 73.83 | 72.70 | 46.07 | 90.65 | 0.11 | 8.06 | 240 | 75.08 | 74.42 | 53.53 | 88.59 | 0.09 | 6.71 | 16 | 79.91 | 78.66 | 62.02 | 87.66 | 0.09 | 6.84 |
|  | Early Medieval | 114 | 73.94 | 73.67 | 55.59 | 86.44 | 0.08 | 6.24 | 152 | 74.65 | 74.75 | 49.74 | 86.84 | 0.08 | 5.78 |  |  |  |  |  |  |  |
|  | Iron/Roman | 131 | 73.83 | 72.94 | 47.74 | 89.90 | 0.10 | 7.49 | 139 | 74.92 | 74.50 | 55.30 | 87.28 | 0.09 | 6.43 |  |  |  |  |  |  |  |
|  | Bronze | 85 | 74.70 | 74.25 | 46.20 | 87.17 | 0.10 | 7.12 | 114 | 77.34 | 76.74 | 58.08 | 88.91 | 0.07 | 5.38 | 10 | 76.11 | 76.32 | 67.11 | 84.17 | 0.08 | 5.94 |
|  | Neolithic | 119 | 75.74 | 75.12 | 46.49 | 88.96 | 0.11 | 8.19 | 184 | 78.12 | 78.38 | 61.83 | 99.97 | 0.08 | 6.30 | 29 | 78.56 | 77.99 | 51.05 | 99.24 | 0.13 | 9.81 |
|  | Mesolithic | 14 | 83.15 | 79.77 | 58.06 | 89.73 | 0.10 | 8.24 | 36 | 77.68 | 78.81 | 66.16 | 88.33 | 0.07 | 5.21 |  |  |  |  |  |  |  |
|  | Upper Paleolithic | 16 | 79.82 | 80.75 | 69.40 | 93.42 | 0.09 | 7.07 | 26 | 80.37 | 80.22 | 68.62 | 91.54 | 0.07 | 5.76 |  |  |  |  |  |  |  |
|  | Middle Paleolithic | 2 | 78.54 | 78.54 | 76.04 | 81.04 | 0.04 | 3.53 | 3 | 78.26 | 76.47 | 69.08 | 82.06 | 0.09 | 6.68 | 1 | 79.37 | 79.37 | 79.37 | 79.37 | 0.00 | 0.00 |
|  | Ix/Iy |  |  |  |  |  |  |  |  |  |  |  |  |  |  |  |  |  |  |  |  |  |
|  | Very recent | 57 | 0.96 | 1.00 | 0.52 | 1.51 | 0.22 | 0.22 | 94 | 0.97 | 1.01 | 0.52 | 1.78 | 0.21 | 0.21 |  |  |  |  |  |  |  |
|  | Early modern | 55 | 0.90 | 0.92 | 0.50 | 1.52 | 0.23 | 0.21 | 102 | 0.99 | 1.02 | 0.58 | 1.76 | 0.24 | 0.24 | 3 | 1.37 | 1.29 | 1.08 | 1.42 | 0.18 | 0.18 |
|  | Late Medieval | 217 | 0.95 | 0.97 | 0.38 | 1.62 | 0.21 | 0.20 | 240 | 1.00 | 1.02 | 0.63 | 1.55 | 0.19 | 0.19 | 16 | 0.97 | 0.94 | 0.69 | 1.13 | 0.16 | 0.15 |
|  | Early Medieval | 114 | 0.92 | 0.94 | 0.62 | 1.35 | 0.17 | 0.16 | 152 | 0.98 | 1.01 | 0.56 | 1.72 | 0.21 | 0.21 |  |  |  |  |  |  |  |
|  | Iron/Roman | 131 | 0.86 | 0.89 | 0.51 | 1.45 | 0.21 | 0.19 | 139 | 0.92 | 0.95 | 0.51 | 1.64 | 0.24 | 0.23 |  |  |  |  |  |  |  |
|  | Bronze | 85 | 0.98 | 0.97 | 0.60 | 1.46 | 0.19 | 0.18 | 114 | 1.05 | 1.06 | 0.62 | 1.84 | 0.19 | 0.20 | 10 | 0.95 | 0.98 | 0.73 | 1.26 | 0.18 | 0.18 |
|  | Neolithic | 119 | 0.93 | 0.95 | 0.59 | 1.49 | 0.18 | 0.17 | 184 | 1.02 | 1.06 | 0.55 | 1.82 | 0.21 | 0.22 | 29 | 1.01 | 1.00 | 0.64 | 1.28 | 0.19 | 0.19 |
|  | Mesolithic | 14 | 1.11 | 1.12 | 0.82 | 1.45 | 0.15 | 0.17 | 36 | 1.17 | 1.21 | 0.82 | 2.04 | 0.24 | 0.29 |  |  |  |  |  |  |  |
|  | Upper Paleolithic | 16 | 1.18 | 1.23 | 0.86 | 1.75 | 0.19 | 0.23 | 26 | 1.46 | 1.45 | 0.91 | 1.91 | 0.21 | 0.30 |  |  |  |  |  |  |  |
|  | Middle Paleolithic | 2 | 1.38 | 1.38 | 1.32 | 1.44 | 0.07 | 0.09 | 3 | 1.81 | 1.71 | 1.43 | 1.88 | 0.14 | 0.24 | 1 | 1.59 | 1.59 | 1.59 | 1.59 | 0.00 | 0.00 |

|  | Age | | | | | | | | | | | | | | | | | | |  |  |  |
| --- | --- | --- | --- | --- | --- | --- | --- | --- | --- | --- | --- | --- | --- | --- | --- | --- | --- | --- | --- | --- | --- | --- |
|  | Very recent | 57 | 40.00 | 42.78 | 20.00 | 87.00 | 0.37 | 15.81 | 94 | 39.00 | 42.24 | 18.00 | 81.00 | 0.36 | 15.02 |  |  |  |  |  |  |  |
|  | Early modern | 55 | 37.00 | 38.99 | 21.50 | 80.00 | 0.30 | 11.84 | 102 | 39.50 | 40.21 | 21.00 | 80.00 | 0.29 | 11.67 | 3 | 38.00 | 38.00 | 38.00 | 38.00 | 0.00 | 0.00 |
|  | Late Medieval | 217 | 35.00 | 23.00 | 19.00 | 65.00 | 0.45 | 10.46 | 240 | 37.25 | 35.83 | 19.50 | 65.00 | 0.30 | 10.62 | 16 | 38.00 | 38.00 | 38.00 | 38.00 | 0.00 | 0.00 |
|  | Early Medieval | 114 | 35.00 | 38.07 | 19.00 | 65.00 | 0.28 | 10.84 | 152 | 42.50 | 41.05 | 21.00 | 65.00 | 0.24 | 9.89 |  |  |  |  |  |  |  |
|  | Iron/Roman | 131 | 35.00 | 35.28 | 20.00 | 65.00 | 0.31 | 10.85 | 139 | 40.00 | 38.94 | 18.00 | 65.00 | 0.29 | 11.12 |  |  |  |  |  |  |  |
|  | Bronze | 85 | 35.00 | 37.70 | 19.00 | 65.00 | 0.26 | 9.74 | 114 | 38.75 | 38.03 | 18.00 | 65.00 | 0.26 | 9.74 | 10 | 38.00 | 38.00 | 38.00 | 38.00 | 0.00 | 0.00 |
|  | Neolithic | 119 | 35.00 | 35.03 | 19.00 | 65.00 | 0.33 | 11.49 | 184 | 37.25 | 35.44 | 19.00 | 55.00 | 0.26 | 9.39 | 29 | 38.00 | 38.00 | 38.00 | 38.00 | 0.00 | 0.00 |
|  | Mesolithic | 14 | 38.00 | 41.32 | 20.00 | 55.00 | 0.25 | 10.23 | 36 | 38.00 | 36.61 | 17.00 | 55.00 | 0.22 | 8.03 |  |  |  |  |  |  |  |
|  | Upper Paleolithic | 16 | 32.50 | 31.34 | 19.00 | 38.00 | 0.23 | 7.15 | 26 | 38.00 | 38.12 | 18.00 | 55.00 | 0.20 | 7.66 |  |  |  |  |  |  |  |
|  | Middle Paleolithic | 2 | 23.00 | 23.00 | 18.50 | 27.50 | 0.28 | 6.36 | 3 | 35.00 | 35.83 | 27.50 | 45.00 | 0.25 | 8.78 | 1 | 30.00 | 30.00 | 30.00 | 30.00 | 0.00 | 0.00 |

Abbreviations: Period = division into archaeologically/historically temporal periods according to Ruff (2018), except for Paleolithic age were other classification was used; min = the smallest valued; max = the largest value; CV = coefficient of variation; SD = standard deviation; J = standardized polar moment of area (index of femoral robusticity); %CA = percent of cortical area in a cross section; Ix/Iy – ratio of Ix and Iy; Age = mean age of each individual.

**SOM Table S4**

Descriptive statistics of the femoral biomechanical properties and age of Neandertals according to sex

|  | Female | | | | | | | Male | | | | | | |
| --- | --- | --- | --- | --- | --- | --- | --- | --- | --- | --- | --- | --- | --- | --- |
| Variable | n | Median | Mean | Min | Max | CV | SD | *n* | Median | Mean | Min | Max | CV | SD |
| J | 4 | 1808.71 | 1782.04 | 1352.95 | 2157.79 | 0.21 | 378.17 | 9 | 2270.96 | 2314.27 | 1657.43 | 3291.93 | 0.21 | 476.80 |
| %CA | 4 | 76.13 | 76.09 | 66.20 | 85.92 | 0.11 | 8.06 | 9 | 79.73 | 81.40 | 76.26 | 90.98 | 0.06 | 4.87 |
| Ix/Iy | 4 | 0.85 | 0.83 | 0.70 | 0.94 | 0.13 | 0.11 | 9 | 0.93 | 0.95 | 0.88 | 1.24 | 0.15 | 0.14 |
| Age | 4 | 25.75 | 24.88 | 18.00 | 30.00 | 0.21 | 5.25 | 9 | 28.75 | 32.46 | 23.00 | 50.00 | 0.26 | 8.39 |

Abbreviations: min = the smallest valued; max = the largest value; CV = coefficient of variation; SD = standard deviation; J = standardized polar moment of area (index of femoral robusticity); %CA = percent of cortical area in a cross section; Ix/Iy = ratio of Ix and Iy; age =mean age of each individual.

**SOM Table S5**

The Akaike Information Criteria for particular models for *Homo sapiens* and Neandertals

|  | Predictors | Df | logLik | AIC | Delta AIC | Weight |
| --- | --- | --- | --- | --- | --- | --- |
| *Homo sapiens* | | | | | | |
| 1 | Years (BP)+ Lifestyle+ Sex+ Age+%CA+ Ix/Iy+ Maximum slope | 7 | -14378.67 | 28773.3 | 0 | 1 |
| 2 | Years (BP)+ Lifestyle+ Sex+ Age+%CA+ Ix/Iy | 6 | -14412.40 | 28838.8 | 65.46 | 0 |
| 3 | Years (BP)+ Lifestyle+ Sex+ Age+%CA | 5 | -14423.60 | 28859.2 | 85.85 | 0 |
| 4 | Years (BP)+ Lifestyle+ Sex+ Age | 4 | -14428.41 | 28866.8 | 93.47 | 0 |
| 5 | Years (BP)+ Lifestyle+ Sex | 3 | -14594.48 | 29197.0 | 423.62 | 0 |
| 6 | Years (BP)+ Lifestyle | 2 | -14599.16 | 29204.3 | 430.98 | 0 |
| 7 | Years (BP) | 2 | -14610.39 | 29224.8 | 451.44 | 0 |
| Neandertals | | | | | | |
| 1 | Years (BP)+ Sex+ %CA+ Ix/Iy+ Maximum slope | 10 | -65.362 | 150.7 | 0 | 0.999 |
| 2 | Years (BP)+ Sex+ %CA+ Ix/Iy | 9 | -73.578 | 165.2 | 14.43 | 0.001 |
| 3 | Years (BP)+ Sex+%CA | 8 | -77.969 | 171.9 | 21.21 | 0 |
| 4 | Years (BP)+ Sex | 7 | -83.984 | 182.0 | 182.0 | 0 |
| 5 | Years (BP)+ Sex | 6 | -92.540 | 197.1 | 46.36 | 0 |

Df = degrees of freedom; logLik = log-Likelihood; AIC = Akaike Information Criterion; Delta AIC = Delta Akaike Information Criterion; Weight = Akaike Information Criterion weights; Age = mean age of each individual; %CA = percent of cortical area in a cross section; Ix/Iy – ratio of Ix and Iy
